# Chlorido[2-(diphenyl­phos­phino)­aceto­phenone]gold(I)

**DOI:** 10.1107/S1600536807066299

**Published:** 2007-12-18

**Authors:** Uwe Monkowius, Manfred Zabel

**Affiliations:** a Johannes Kepler Universität Linz, Institut für Anorganische Chemie, Altenbergerstrasse 69, 4040 Linz, Austria; b Universität Regensburg, Zentrale Analytik, Röntgenstrukturanalyse, Universitätsstrasse 31, 93053 Regensburg, Germany

## Abstract

In the crystal structure of the title compound, [AuCl(C_20_H_17_OP)], the phosphine acts as a monodentate ligand. The Au atoms are attached solely to the P and Cl atoms. The coordination is linear without any tendency to aggregate *via* aurophilic inter­actions.

## Related literature

For related literature, see: Monkowius *et al.* (2003*a* ▶,*b* ▶); Coote *et al.* (1993 ▶); Johansson *et al.* (2002 ▶).
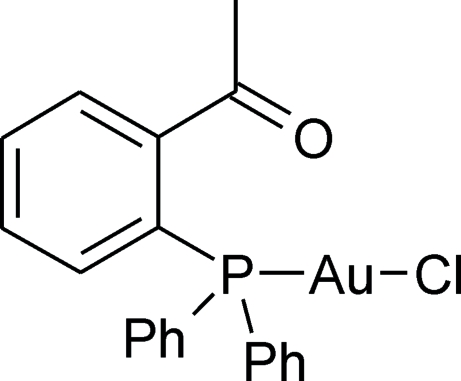


## Experimental

### 

#### Crystal data

[AuCl(C_20_H_17_OP)]
*M* *_r_* = 536.73
Monoclinic, 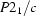

*a* = 11.3665 (8) Å
*b* = 9.3110 (9) Å
*c* = 18.7813 (14) Å
β = 103.945 (8)°
*V* = 1929.1 (3) Å^3^
*Z* = 4
Mo *K*α radiation
μ = 7.85 mm^−1^
*T* = 297 K
0.42 × 0.12 × 0.04 mm

#### Data collection

Stoe IPDS diffractometer
Absorption correction: analytical (*X-SHAPE* and *X-RED*; Stoe, 1998 ▶) *T*_min_ = 0.029, *T*_max_ = 0.099
21230 measured reflections
4045 independent reflections
3224 reflections with *I* > 2σ(*I*)
*R*_int_ = 0.078

#### Refinement

*R*[*F* ^2^ > 2σ(*F* ^2^)] = 0.042
*wR*(*F* ^2^) = 0.106
*S* = 0.96
4045 reflections
217 parameters
H-atom parameters constrained
Δρ_max_ = 2.24 e Å^−3^
Δρ_min_ = −0.85 e Å^−3^

### 

Data collection: *IPDS Software* (Stoe, 1998 ▶); cell refinement: *IPDS Software*; data reduction: *IPDS Software*; program(s) used to solve structure: *SIR97* (Altomare *et al*., 1999 ▶); program(s) used to refine structure: *SHELXL97* (Sheldrick, 1997 ▶); molecular graphics: *PLATON* (Spek, 2003 ▶); software used to prepare material for publication: *PLATON*.

## Supplementary Material

Crystal structure: contains datablocks global, I. DOI: 10.1107/S1600536807066299/zl2093sup1.cif

Structure factors: contains datablocks I. DOI: 10.1107/S1600536807066299/zl2093Isup2.hkl

Additional supplementary materials:  crystallographic information; 3D view; checkCIF report

## Figures and Tables

**Fig. 1.**
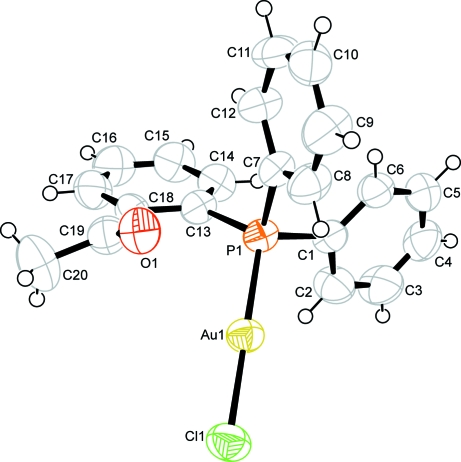
View of the title compound with the atom numbering scheme. Displacement ellipsoids for non-H atoms are drawn at the 50% probability level.

## supplementary crystallographic information

### Comment

The title compound was prepared from 2-(diphenylphosphino)acetophenone and (tht)AuCl (tht = tetrahydrothiophene) in methylene chloride in nearly quantitative yields. The crystals are built of monomeric units which show no tendency to aggregate *via* aurophilic interactions. In principle, the applied phosphine is capable of coordinating as a bidentate *P*,*O*-ligand (Johansson *et al.*, 2002). Nevertheless, the gold atom is in a standard linear coordination [P—Au—Cl 178.99 (6)°] and entertains no short oxygen contacts. The Au—Cl and Au—P bond lengths are 2.2838 (19) and 2.2323 (16) Å, respectively, and are lying in the expected range for (aryl_3_P)AuCl complexes (Monkowius *et al.*, 2003*a*,b). All Au—P—C angles are larger than the tetrahedral standard [109.9 (2)–116.0 (2)°], and all C—P—C angles are smaller [103.8 (3)–106.4°]. The keto group of the phosphine ligand is twisted out of the plane of the aromatic ring by 26.5 (10)° (C13—C18—C19—O1), with the oxygen oriented towards the gold atom.

### Experimental

2-(Diphenylphosphino)acetophenone was synthesized according to a published procedure (Coote *et al.*, 1993). The title complex was prepared analogous to a previously published procedure (Monkowius *et al.*, 2003*a*,b): 2-(diphenylphosphino)acetophenone (0.10 g, 0.31 mmol) and (tht)AuCl (0.10 g, 0.31 mmol, tht = tetrahydrothiophene) were stirred in methylene chloride (20 ml) at room temperature for 2 h. The product was precipitated with *n*-pentane and isolated by filtration. Recrystallization from methylene chloride/diethylether yields colourless crystals suitable for X-ray crystallography. Yield: 0.16 mg (0.30 mmol, 97%); ^1^H-NMR (300 MHz, CDCl_3_): δ = 8.09 (ddd, J = 7.75, 4.42, 1.17 Hz, 1 H, C_6_H_4_), 7.69 (*pseudo*-tt, J = 7.75, 1.32 Hz, 1 H, C_6_H_4_), 7.39–7.56 (m, 11 H, Ph_2_P, C_6_H_4_), 6.99 (ddd, J = 12.91, 1.05, 1.08 Hz, 1 H, C_6_H_4_), 2.59 (s, 3 H, CH_3_).

### Refinement

The data were collected at room temperature. The structure was solved by direct methods (*SIR97*) and refined by full-matrix anisotropic least squares (*SHELXL97*). The H-atoms were calculated geometrically and a riding model was used during refinement process.

### Crystal data

**Table:**

[AuCl(C_20_H_17_OP)]	*F*_000_ = 1024
*M**_r_* = 536.73	Cell parameters were determined by indexing 8000 reflections with I/sigma limit 6.0.
Monoclinic, *P*2_1_/*c*	*D*_x_ = 1.848 Mg m^−^^3^
Hall symbol: -P 2ybc	Mo *K*α radiation λ = 0.71073 Å
*a* = 11.3665 (8) Å	Cell parameters from 8000 reflections
*b* = 9.3110 (9) Å	θ = 2.2–26.8º
*c* = 18.7813 (14) Å	µ = 7.85 mm^−^^1^
β = 103.945 (8)º	*T* = 297 K
*V* = 1929.1 (3) Å^3^	Rod, colourless
*Z* = 4	0.42 × 0.12 × 0.04 mm

### Data collection

**Table:**

Stoe IPDS diffractometer	4045 independent reflections
Radiation source: fine-focus sealed tube	3224 reflections with *I* > 2σ(*I*)
Monochromator: graphite	*R*_int_ = 0.078
*T* = 297(1) K	θ_max_ = 26.8º
rotation scans	θ_min_ = 2.2º
Absorption correction: analytical(X-SHAPE and X-RED; Stoe, 1998)	*h* = −14→14
*T*_min_ = 0.029, *T*_max_ = 0.099	*k* = −11→11
21230 measured reflections	*l* = −23→23

### Refinement

**Table:**

Refinement on *F*^2^	Secondary atom site location: difference Fourier map
Least-squares matrix: full	Hydrogen site location: inferred from neighbouring sites
*R*[*F*^2^ > 2σ(*F*^2^)] = 0.042	H-atom parameters constrained
*wR*(*F*^2^) = 0.106	*w* = 1/[σ^2^(*F*_o_^2^) + (0.0744*P*)^2^] where *P* = (*F*_o_^2^ + 2*F*_c_^2^)/3
*S* = 0.96	(Δ/σ)_max_ = 0.001
4045 reflections	Δρ_max_ = 2.24 e Å^−^^3^
217 parameters	Δρ_min_ = −0.84 e Å^−^^3^
Primary atom site location: structure-invariant direct methods	Extinction correction: none

### Special details

**Table:**

Experimental. Data were collected applying an imaging plate system (Stoe) with the following measurement parameters:Detector distance [mm] 65 Phi movement mode Oscillation Phi incr. [degrees] 1.0 Number of exposures 260 Irradiation / exposure [min] 5.00For a detailed description of the method see: Sheldrick, G.*M*., Paulus, E. Vertesy, *L*. & Hahn, F. (1995) Acta Cryst. B51, 89–98.
Geometry. Bond distances, angles *etc*. have been calculated using the rounded fractional coordinates. All su's are estimated from the variances of the (full) variance-covariance matrix. The cell e.s.d.'s are taken into account in the estimation of distances, angles and torsion angles
Refinement. Refinement of *F*^2^ against ALL reflections. The weighted *R*-factor *wR* and goodness of fit *S* are based on *F*^2^, conventional *R*-factors *R* are based on *F*, with *F* set to zero for negative *F*^2^. The threshold expression of *F*^2^ > σ(*F*^2^) is used only for calculating *R*-factors(gt) *etc*. and is not relevant to the choice of reflections for refinement. *R*-factors based on *F*^2^ are statistically about twice as large as those based on *F*, and *R*- factors based on ALL data will be even larger.

### Fractional atomic coordinates and isotropic or equivalent isotropic displacement parameters (Å^2^)

**Table:**

	*x*	*y*	*z*	*U*_iso_*/*U*_eq_	
Au1	0.83069 (2)	0.15157 (2)	0.39235 (1)	0.0549 (1)	
Cl1	0.96075 (16)	0.00052 (19)	0.35351 (11)	0.0735 (6)	
P1	0.70126 (14)	0.29885 (16)	0.42840 (8)	0.0509 (4)	
O1	0.7785 (5)	0.4367 (5)	0.3125 (3)	0.0733 (17)	
C1	0.6561 (6)	0.2224 (6)	0.5065 (3)	0.0562 (17)	
C2	0.7470 (8)	0.1689 (8)	0.5639 (5)	0.078 (3)	
C3	0.7186 (10)	0.1166 (10)	0.6266 (5)	0.093 (3)	
C4	0.6024 (10)	0.1173 (9)	0.6333 (5)	0.083 (3)	
C5	0.5085 (8)	0.1650 (7)	0.5755 (5)	0.077 (3)	
C6	0.5369 (6)	0.2179 (7)	0.5125 (4)	0.065 (2)	
C7	0.5599 (6)	0.3296 (5)	0.3609 (3)	0.0543 (17)	
C8	0.5156 (7)	0.2220 (7)	0.3111 (4)	0.071 (2)	
C9	0.4060 (8)	0.2378 (9)	0.2603 (5)	0.091 (3)	
C10	0.3411 (7)	0.3604 (8)	0.2587 (5)	0.078 (3)	
C11	0.3850 (7)	0.4692 (8)	0.3066 (4)	0.078 (3)	
C12	0.4945 (6)	0.4552 (7)	0.3587 (4)	0.068 (2)	
C13	0.7607 (5)	0.4768 (6)	0.4609 (3)	0.0554 (17)	
C14	0.7386 (6)	0.5287 (8)	0.5259 (4)	0.067 (2)	
C15	0.7808 (8)	0.6639 (7)	0.5525 (5)	0.078 (3)	
C16	0.8470 (7)	0.7445 (9)	0.5154 (5)	0.085 (3)	
C17	0.8714 (7)	0.6944 (8)	0.4532 (5)	0.077 (3)	
C18	0.8290 (5)	0.5625 (7)	0.4224 (4)	0.0603 (19)	
C19	0.8492 (6)	0.5158 (8)	0.3518 (4)	0.069 (2)	
C20	0.9575 (9)	0.5755 (13)	0.3279 (6)	0.107 (4)	
H2	0.82700	0.16840	0.56000	0.0940*	
H3	0.77980	0.08050	0.66460	0.1110*	
H4	0.58500	0.08580	0.67670	0.0990*	
H5	0.42840	0.16130	0.57920	0.0930*	
H6	0.47530	0.25080	0.47390	0.0780*	
H8	0.56000	0.13790	0.31180	0.0850*	
H9	0.37690	0.16440	0.22710	0.1090*	
H10	0.26680	0.37040	0.22500	0.0940*	
H11	0.34100	0.55390	0.30430	0.0930*	
H12	0.52320	0.52930	0.39150	0.0810*	
H14	0.69530	0.47280	0.55180	0.0800*	
H15	0.76390	0.69900	0.59530	0.0930*	
H16	0.87530	0.83450	0.53310	0.1010*	
H17	0.91860	0.75030	0.42980	0.0930*	
H20A	1.03060	0.54920	0.36300	0.1280*	
H20B	0.95140	0.67820	0.32470	0.1280*	
H20C	0.95910	0.53680	0.28070	0.1280*	

### Atomic displacement parameters (Å^2^)

**Table:**

	*U*^11^	*U*^22^	*U*^33^	*U*^12^	*U*^13^	*U*^23^
Au1	0.0536 (2)	0.0529 (2)	0.0568 (2)	0.0033 (1)	0.0104 (1)	−0.0045 (1)
Cl1	0.0653 (9)	0.0705 (9)	0.0882 (12)	0.0053 (7)	0.0251 (9)	−0.0151 (8)
P1	0.0534 (8)	0.0480 (7)	0.0485 (7)	0.0022 (6)	0.0068 (6)	−0.0027 (6)
O1	0.081 (3)	0.081 (3)	0.059 (3)	−0.008 (3)	0.019 (2)	−0.005 (2)
C1	0.062 (3)	0.051 (3)	0.056 (3)	0.005 (3)	0.015 (3)	0.002 (2)
C2	0.071 (5)	0.096 (5)	0.065 (4)	0.014 (4)	0.014 (4)	0.018 (4)
C3	0.103 (7)	0.104 (6)	0.072 (5)	0.026 (5)	0.022 (5)	0.035 (4)
C4	0.104 (7)	0.078 (4)	0.073 (5)	0.005 (4)	0.034 (5)	0.020 (4)
C5	0.075 (5)	0.074 (4)	0.089 (5)	−0.001 (3)	0.033 (4)	0.008 (4)
C6	0.067 (4)	0.062 (3)	0.066 (4)	0.007 (3)	0.017 (3)	0.008 (3)
C7	0.062 (3)	0.047 (3)	0.052 (3)	0.001 (2)	0.010 (3)	0.003 (2)
C8	0.077 (4)	0.052 (3)	0.075 (4)	−0.001 (3)	0.001 (3)	−0.008 (3)
C9	0.089 (5)	0.072 (4)	0.089 (5)	−0.013 (4)	−0.021 (4)	−0.007 (4)
C10	0.062 (4)	0.090 (5)	0.072 (5)	−0.006 (3)	−0.004 (4)	0.017 (4)
C11	0.075 (4)	0.074 (4)	0.080 (5)	0.029 (4)	0.012 (4)	0.019 (4)
C12	0.072 (4)	0.059 (3)	0.066 (4)	0.012 (3)	0.005 (3)	−0.002 (3)
C13	0.056 (3)	0.051 (3)	0.053 (3)	−0.001 (2)	0.001 (3)	−0.004 (2)
C14	0.068 (4)	0.069 (4)	0.062 (4)	−0.004 (3)	0.012 (3)	−0.013 (3)
C15	0.078 (5)	0.074 (4)	0.076 (5)	−0.008 (4)	0.009 (4)	−0.028 (3)
C16	0.082 (5)	0.066 (4)	0.097 (6)	−0.011 (4)	0.004 (4)	−0.025 (4)
C17	0.062 (4)	0.065 (4)	0.102 (6)	−0.012 (3)	0.014 (4)	−0.012 (4)
C18	0.043 (3)	0.063 (3)	0.071 (4)	−0.005 (3)	0.006 (3)	0.000 (3)
C19	0.064 (4)	0.071 (4)	0.068 (4)	0.003 (3)	0.010 (3)	0.009 (3)
C20	0.099 (7)	0.134 (9)	0.101 (7)	−0.026 (6)	0.050 (6)	−0.014 (6)

### Geometric parameters (Å, °)

**Table:**

Au1—Cl1	2.2838 (19)	C16—C17	1.347 (13)
Au1—P1	2.2323 (16)	C17—C18	1.393 (10)
P1—C1	1.812 (6)	C18—C19	1.465 (10)
P1—C7	1.814 (6)	C19—C20	1.514 (13)
P1—C13	1.837 (6)	C2—H2	0.9300
O1—C19	1.203 (9)	C3—H3	0.9300
C1—C2	1.393 (11)	C4—H4	0.9300
C1—C6	1.387 (10)	C5—H5	0.9300
C2—C3	1.383 (13)	C6—H6	0.9300
C3—C4	1.357 (16)	C8—H8	0.9300
C4—C5	1.399 (13)	C9—H9	0.9300
C5—C6	1.390 (11)	C10—H10	0.9300
C7—C8	1.380 (9)	C11—H11	0.9300
C7—C12	1.381 (9)	C12—H12	0.9300
C8—C9	1.382 (12)	C14—H14	0.9300
C9—C10	1.356 (12)	C15—H15	0.9300
C10—C11	1.367 (11)	C16—H16	0.9300
C11—C12	1.392 (11)	C17—H17	0.9300
C13—C14	1.391 (9)	C20—H20A	0.9600
C13—C18	1.426 (9)	C20—H20B	0.9600
C14—C15	1.396 (10)	C20—H20C	0.9600
C15—C16	1.367 (12)		
			
Au1···O1	3.037 (5)	C13···H12	2.7400
Au1···C19	3.493 (7)	C14···H20A^i^	3.0200
Au1···C16^i^	3.782 (8)	C14···H12	3.0600
Au1···C10^ii^	4.067 (8)	C15···H20A^i^	3.0700
Au1···C17^i^	4.148 (9)	C15···H6^viii^	2.9400
Au1···H2	3.1600	C17···H20B	2.7800
Au1···H10^ii^	3.4300	C17···H20A	3.0700
Au1···H8	3.0900	C19···H9^vi^	2.9800
Au1···H16^i^	3.3000	C20···H17	2.6300
Cl1···H17^iii^	2.8400	H2···Au1	3.1600
Cl1···H20B^iii^	3.0500	H2···Cl1^v^	3.0100
Cl1···H10^ii^	2.9100	H3···Cl1^v^	3.1400
Cl1···H20C^iv^	2.9000	H3···O1^ix^	2.7900
Cl1···H2^v^	3.0100	H6···C7	2.6400
Cl1···H3^v^	3.1400	H6···C12	2.9300
Cl1···H16^i^	2.9100	H6···C15^viii^	2.9400
P1···O1	2.842 (6)	H8···Au1	3.0900
O1···Au1	3.037 (5)	H8···C11^ii^	2.9100
O1···P1	2.842 (6)	H9···O1^ii^	2.7400
O1···C7	3.014 (9)	H9···C19^ii^	2.9800
O1···H9^vi^	2.7400	H10···Au1^vi^	3.4300
O1···H3^vii^	2.7900	H10···Cl1^vi^	2.9100
C2···C14	3.422 (11)	H12···C13	2.7400
C6···C12	3.576 (10)	H12···C14	3.0600
C7···O1	3.014 (9)	H12···C5^viii^	2.9500
C8···C11^ii^	3.593 (10)	H14···C1	2.4900
C10···Au1^vi^	4.067 (8)	H14···C2	2.8900
C11···C8^vi^	3.593 (10)	H14···C6	2.9600
C12···C6	3.576 (10)	H14···C11^viii^	3.0600
C14···C2	3.422 (11)	H16···Au1^i^	3.3000
C16···Au1^i^	3.782 (8)	H16···Cl1^i^	2.9100
C17···Au1^i^	4.148 (9)	H17···Cl1^x^	2.8400
C19···Au1	3.493 (7)	H17···C20	2.6300
C1···H14	2.4900	H17···H20B	2.2000
C2···H14	2.8900	H20A···C17	3.0700
C5···H12^viii^	2.9500	H20A···C14^i^	3.0200
C6···H14	2.9600	H20A···C15^i^	3.0700
C7···H6	2.6400	H20B···Cl1^x^	3.0500
C11···H14^viii^	3.0600	H20B···C17	2.7800
C11···H8^vi^	2.9100	H20B···H17	2.2000
C12···H6	2.9300	H20C···Cl1^xi^	2.9000
			
Cl1—Au1—P1	178.99 (6)	C18—C19—C20	118.4 (7)
Au1—P1—C1	109.9 (2)	C1—C2—H2	120.00
Au1—P1—C7	115.00 (19)	C3—C2—H2	120.00
Au1—P1—C13	116.0 (2)	C2—C3—H3	120.00
C1—P1—C7	104.6 (3)	C4—C3—H3	120.00
C1—P1—C13	103.8 (3)	C3—C4—H4	120.00
C7—P1—C13	106.4 (2)	C5—C4—H4	120.00
P1—C1—C2	117.7 (6)	C4—C5—H5	121.00
P1—C1—C6	123.4 (5)	C6—C5—H5	121.00
C2—C1—C6	118.9 (6)	C1—C6—H6	120.00
C1—C2—C3	120.2 (9)	C5—C6—H6	120.00
C2—C3—C4	120.6 (9)	C7—C8—H8	120.00
C3—C4—C5	120.5 (9)	C9—C8—H8	120.00
C4—C5—C6	118.9 (8)	C8—C9—H9	120.00
C1—C6—C5	120.8 (7)	C10—C9—H9	120.00
P1—C7—C8	118.4 (5)	C9—C10—H10	120.00
P1—C7—C12	122.3 (4)	C11—C10—H10	120.00
C8—C7—C12	119.3 (6)	C10—C11—H11	119.00
C7—C8—C9	120.8 (7)	C12—C11—H11	119.00
C8—C9—C10	120.0 (8)	C7—C12—H12	121.00
C9—C10—C11	119.9 (8)	C11—C12—H12	121.00
C10—C11—C12	121.1 (7)	C13—C14—H14	120.00
C7—C12—C11	119.0 (6)	C15—C14—H14	120.00
P1—C13—C14	118.4 (5)	C14—C15—H15	120.00
P1—C13—C18	122.5 (4)	C16—C15—H15	120.00
C14—C13—C18	119.1 (6)	C15—C16—H16	120.00
C13—C14—C15	120.6 (7)	C17—C16—H16	120.00
C14—C15—C16	119.8 (8)	C16—C17—H17	119.00
C15—C16—C17	120.4 (8)	C18—C17—H17	119.00
C16—C17—C18	122.8 (8)	C19—C20—H20A	110.00
C13—C18—C17	117.3 (6)	C19—C20—H20B	109.00
C13—C18—C19	121.1 (6)	C19—C20—H20C	109.00
C17—C18—C19	121.6 (6)	H20A—C20—H20B	110.00
O1—C19—C18	120.7 (6)	H20A—C20—H20C	109.00
O1—C19—C20	120.9 (7)	H20B—C20—H20C	109.00
			
Au1—P1—C1—C2	47.5 (5)	C4—C5—C6—C1	0.6 (10)
C7—P1—C1—C2	171.4 (5)	P1—C7—C8—C9	177.6 (6)
C13—P1—C1—C2	−77.2 (6)	C8—C7—C12—C11	0.6 (10)
Au1—P1—C1—C6	−134.2 (5)	C12—C7—C8—C9	−1.2 (11)
C7—P1—C1—C6	−10.2 (6)	P1—C7—C12—C11	−178.2 (5)
C13—P1—C1—C6	101.2 (5)	C7—C8—C9—C10	0.4 (13)
Au1—P1—C13—C14	−133.4 (5)	C8—C9—C10—C11	1.0 (13)
Au1—P1—C7—C8	29.6 (6)	C9—C10—C11—C12	−1.7 (13)
C1—P1—C7—C8	−91.0 (6)	C10—C11—C12—C7	0.9 (11)
C13—P1—C7—C8	159.5 (5)	P1—C13—C14—C15	−179.4 (6)
Au1—P1—C7—C12	−151.7 (5)	C18—C13—C14—C15	1.1 (10)
C1—P1—C7—C12	87.7 (6)	P1—C13—C18—C17	−178.7 (5)
C13—P1—C7—C12	−21.8 (6)	P1—C13—C18—C19	3.7 (9)
C7—P1—C13—C14	97.3 (5)	C14—C13—C18—C17	0.8 (9)
Au1—P1—C13—C18	46.2 (5)	C14—C13—C18—C19	−176.8 (6)
C1—P1—C13—C14	−12.7 (6)	C13—C14—C15—C16	−1.7 (12)
C7—P1—C13—C18	−83.2 (5)	C14—C15—C16—C17	0.2 (13)
C1—P1—C13—C18	166.8 (5)	C15—C16—C17—C18	1.8 (13)
C6—C1—C2—C3	−2.0 (11)	C16—C17—C18—C13	−2.3 (11)
P1—C1—C2—C3	176.4 (6)	C16—C17—C18—C19	175.3 (8)
C2—C1—C6—C5	1.9 (10)	C13—C18—C19—O1	26.5 (10)
P1—C1—C6—C5	−176.5 (5)	C13—C18—C19—C20	−156.6 (7)
C1—C2—C3—C4	−0.4 (13)	C17—C18—C19—O1	−150.9 (7)
C2—C3—C4—C5	2.9 (13)	C17—C18—C19—C20	26.0 (11)
C3—C4—C5—C6	−3.0 (12)		

Symmetry codes: (i) −*x*+2, −*y*+1, −*z*+1; (ii) −*x*+1, *y*−1/2, −*z*+1/2; (iii) *x*, *y*−1, *z*; (iv) −*x*+2, *y*−1/2, −*z*+1/2; (v) −*x*+2, −*y*, −*z*+1; (vi) −*x*+1, *y*+1/2, −*z*+1/2; (vii) *x*, −*y*+1/2, *z*−1/2; (viii) −*x*+1, −*y*+1, −*z*+1; (ix) *x*, −*y*+1/2, *z*+1/2; (x) *x*, *y*+1, *z*; (xi) −*x*+2, *y*+1/2, −*z*+1/2.
